# Necroptosis Inhibition by Hydrogen Sulfide Alleviated Hypoxia-Induced Cardiac Fibroblasts Proliferation via Sirtuin 3

**DOI:** 10.3390/ijms222111893

**Published:** 2021-11-02

**Authors:** Yue Zhang, Weiwei Gong, Mengting Xu, Shuping Zhang, Jieru Shen, Mingxian Zhu, Yuqin Wang, Yun Chen, Jiahai Shi, Guoliang Meng

**Affiliations:** 1 Department of Pharmacology, School of Pharmacy, Nantong University, Nantong 226001, China; mistyzy@163.com (Y.Z.); m15106292438@163.com (W.G.); xumengting0222@163.com (M.X.); 17826150737@163.com (S.Z.); jieru-shen@outlook.com (J.S.); pangzhuzhu0821@163.com (M.Z.); wangyuqin@ntu.edu.cn (Y.W.); cyun@ntu.edu.cn (Y.C.); 2 Nantong Key Laboratory of Translational Medicine in Cardiothoracic Diseases, Research Institution of Translational Medicine in Cardiothoracic Diseases, Nantong University, Nantong 226001, China

**Keywords:** hydrogen sulfide, hypoxia, cardiac fibroblasts, oxidative stress, sirtuin 3

## Abstract

Myocardial ischemia or hypoxia can induce myocardial fibroblast proliferation and myocardial fibrosis. Hydrogen sulfide (H_2_S) is a gasotransmitter with multiple physiological functions. In our present study, primary cardiac fibroblasts were incubated with H_2_S donor sodium hydrosulfide (NaHS, 50 μM) for 4 h followed by hypoxia stimulation (containing 5% CO_2_ and 1% O_2_) for 4 h. Then, the preventive effects on cardiac fibroblast proliferation and the possible mechanisms were investigated. Our results showed that NaHS reduced the cardiac fibroblast number, decreased the hydroxyproline content; inhibited the EdU positive ratio; and down-regulated the expressions of α-smooth muscle actin (α-SMA), the antigen identified by monoclonal antibody Ki67 (Ki67), proliferating cell nuclear antigen (PCNA), collagen I, and collagen III, suggesting that hypoxia-induced cardiac fibroblasts proliferation was suppressed by NaHS. NaHS improved the mitochondrial membrane potential and attenuated oxidative stress, and inhibited dynamin-related protein 1 (DRP1), but enhanced optic atrophy protein 1 (OPA1) expression. NaHS down-regulated receptor interacting protein kinase 1 (RIPK1) and RIPK3 expression, suggesting that necroptosis was alleviated. NaHS increased the sirtuin 3 (SIRT3) expressions in hypoxia-induced cardiac fibroblasts. Moreover, after SIRT3 siRNA transfection, the inhibitory effects on cardiac fibroblast proliferation, oxidative stress, and necroptosis were weakened. In summary, necroptosis inhibition by exogenous H_2_S alleviated hypoxia-induced cardiac fibroblast proliferation via SIRT3.

## 1. Introduction

Ischemic heart disease is a leading cause of death worldwide, which may lead to severe perioperative ischemia and infarction [1]. Hypoxia, as the common pathophysiological characteristic of ischemic heart diseases, usually induces cellular oxidation and reduction unbalance, mitochondrial oxidative phosphorylation disorder, adenosine triphosphate (ATP) production impairment, and eventually myocardial infarction exacerbation [2]. The damaged tissue due to persistent hypoxia in myocardial infarction can be replaced by fibrous scar with fibroblasts and myofibroblasts. Although the reparative fibrosis is important to prevent ventricular wall rupture, excessive fibrous reaction and reactive fibrosis are prone to induce gradual damage of cardiac function, and ultimately lead to heart failure [3]. Therefore, the prevention of excessive myocardial fibrosis caused by hypoxia is beneficial to cardiac function. However, there is still a lack of effective methods for hypoxia-induced myocardial fibrosis.

Hydrogen sulfide (H_2_S) is an endogenous gasotransmitter molecule with many physiological functions. Our previous studies verified that H_2_S alleviated transverse aortic constriction (TAC) and isoproterenol-induced cardiac hypertrophy [4,5], improved endothelium-dependent aortic vasodilatation in paraquat administrated mice [6], attenuated myocardial remodeling in spontaneously hypertensive rats [7], suppressed diabetes-accelerated atherosclerosis in streptozotocin (STZ)-administrated low density lipoprotein receptor deficiency mice [8], exhibited an anti-atherosclerotic activity in high fat fed apolipoprotein E deficiency mice [9], and protected against myocardial ischemia/reperfusion injury [10]. These data demonstrate the powerful protective effects of H_2_S against cardiovascular diseases. Previous research also found that endogenous H_2_S supplement ameliorated carbon tetrachloride and high fat diet induced liver fibrosis in rats [11]. H_2_S significantly alleviated renal fibrosis by attenuating collagen accumulation and extracellular matrix remodeling in the kidney of diabetic mice and gentamicin-induced rats [12]. H_2_S also prevented smoke-induced pulmonary fibrosis in rats and alleviated systemic sclerosis associated lung fibrosis in mice [13]. These studies suggest that H_2_S was a candidate compound to combat against a variety of pathological fibrosis. However, the protective effect of H_2_S on hypoxia-induced myocardial fibrosis is not clear.

Sirtuin 3 (SIRT3), as a class III histone deacetylase, mainly exists in the mitochondria. SIRT3 reduces the acetylation of lysine to regulate the structure of the target protein, improve mitochondrial function, inhibit reactive oxygen species (ROS), and attenuate oxidative damage [14]. Excessive ROS is prone to trigger necroptosis, which is a newly type of discovered cell death [15]. Receptor interacting protein kinase 1/3 (RIPK1/3) is the key mediator for necroptosis. The combination of RIPK1 and RIPK3 promotes the activation of each other to form a necrotic body, and then activates mixed lineage kinase domain like protein (MLKL) to accelerate cell death [16]. However, whether H_2_S inhibits hypoxia-induced myocardial fibrosis via SIRT3 and necroptosis needs further study.

In our present study, we investigated whether H_2_S inhibited hypoxia-induced cardiac fibroblast proliferation and the possible involvement of SIRT3 from the perspective of oxidative stress and necroptosis. It is beneficial to find novel ideas for preventing against hypoxia-related myocardial fibrosis.

## 2. Results

### 2.1. NaHS Decreases Cell Number and Hydroxyproline Content in Cardiac Fibroblasts with Hypoxia

**Figure 1:**
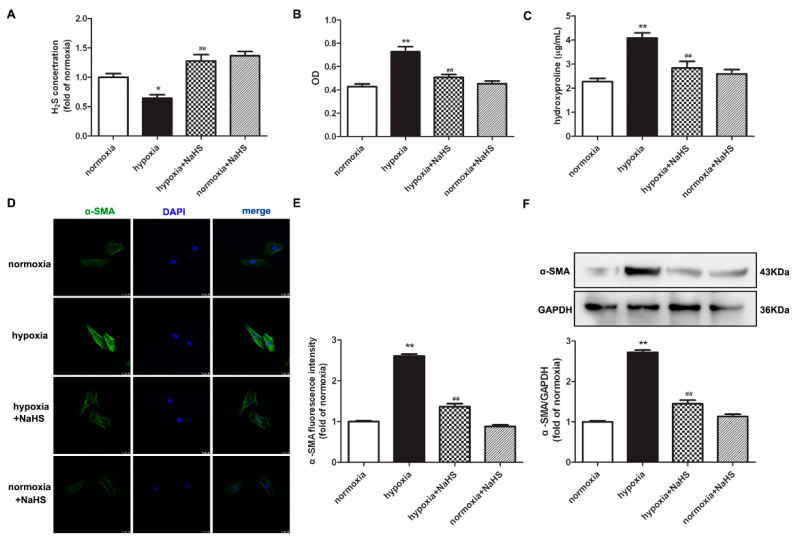
NaHS decreased the cell number, hydroxyproline content, and α-SMA expression in cardiac fibroblasts with hypoxia. After pre-administration with NaHS (50 μM) for 4 h, neonatal rat cardiac fibroblasts were subjected to normoxia (containing 5% CO_2_ and 95% O_2_) or hypoxia (containing 5% CO_2_ and 1% O_2_) for 4 h. (**A**) The H_2_S concentration in the culture medium was measured with an H_2_S-specific microelectrode. (**B**) The cardiac fibroblast number was measured with the CCK-8 kit. (**C**) Hydroxyproline content in the cell culture medium was detected. (**D**) α-SMA was immunofluorescence stained with Alexa Fluor 488 (green)-conjugated IgG. The nuclei were stained with DAPI (blue). Bar = 25 μm. (**E**) The fluorescence intensity of α-SMA was quantified using ImageJ software. (**F**) Expression of α-SMA protein was detected by Western blot. * *p* < 0.05, ** *p* < 0.01 vs. normoxia; ^##^ *p* < 0.01 vs. hypoxia, *n* = 6.

The level of H_2_S in the culture medium was significantly inhibited by hypoxia, which was restored after NaHS pre-administration (Figure 1A). The assessment of the cardiac fibroblast number by cell counting Kit-8 (CCK-8) demonstrated that hypoxia significantly increased the optical density value of the cardiac fibroblasts, which was attenuated by NaHS (Figure 1B). This suggested that NaHS inhibited the hypoxia-induced cardiac fibroblast number. We also found that the concentration of hydroxyproline in the culture medium of cardiac fibroblasts with hypoxia was higher than that with normoxia. NaHS significantly reduced the hydroxyproline content (Figure 1C). These data preliminary showed that NaHS inhibited hypoxia-induced cardiac fibroblast proliferation.

### 2.2. NaHS Suppresses α-SMA Expression in Cardiac Fibroblasts with Hypoxia

Next, α-smooth muscle actin (α-SMA), as a sensitive indicator of myocardial fibroblast proliferation [17], was further detected to evaluate the effect of NaHS on hypoxia-induced cell proliferation. Both immunofluorescence and Western blot found that compared with normoxia, hypoxia elevated the protein expression of α-SMA, which was significantly suppressed by NaHS (Figure 1D–F).

### 2.3. NaHS Inhibits Ki67 and PCNA Expression in Cardiac Fibroblasts with Hypoxia

**Figure 2:**
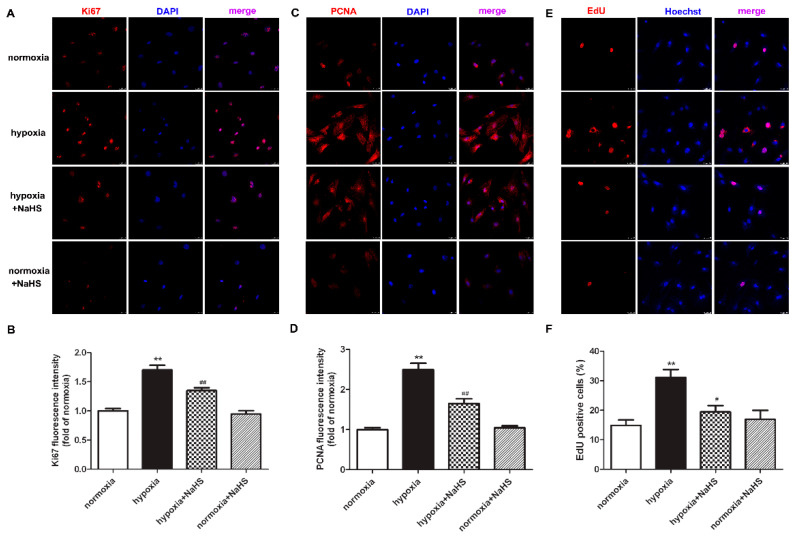
NaHS inhibited the Ki67 and PCNA expression, and reduced the EdU positive cells in the cardiac fibroblasts with hypoxia. After pre-administration with NaHS (50 μM) for 4 h, neonatal rat cardiac fibroblasts were subjected to normoxia (containing 5% CO_2_ and 95% O_2_) or hypoxia (containing 5% CO_2_ and 1% O_2_) for 4 h. (**A**–**D**) Ki67 and PCNA was immunofluorescence stained respectively with Cy3 (red)-conjugated IgG. The nuclei were stained with DAPI (blue). Bar = 25 μm. The fluorescence intensity was quantified using ImageJ software. (**E**) Cell proliferation was assessed by EdU (red) staining. The nuclei were stained with Hoechst (blue). Bar = 25 μm. (**F**) The rate of EdU positive cells was quantified. ** *p* < 0.01 vs. normoxia; ^#^ *p* < 0.05, ^##^ *p* < 0.01 vs. hypoxia, *n* = 6.

Previous studies have demonstrated that the antigen identified by the monoclonal antibody Ki67 is related to cell mitosis, while proliferating cell nuclear antigen (PCNA) plays an important role in the DNA replication processes. That is to say, both Ki67 and PCNA are sensitive indicators of cell proliferation [18,19]. Therefore, the expression of Ki67 and PCNA were also detected to evaluate the effect of NaHS on hypoxia-induced cardiac fibroblast proliferation. Our experimental results found that compared with normoxia, hypoxia up-regulated the expression of Ki67 and PCNA. NaHS significantly inhibited the Ki67 and PCNA expression in the cardiac fibroblast with hypoxia (Figure 2A–D).

### 2.4. NaHS Reduces EdU Positive Cardiac Fibroblasts with Hypoxia

EdU is able to infiltrate the DNA being synthesized, therefore EdU staining is also a common and sensitive means to assess cardiac fibroblast proliferation. Our results showed that compared with normoxia, hypoxia increased EdU positive cardiac fibroblasts. NaHS significantly reduced EdU positive cells in cardiac fibroblasts with hypoxia (Figure 2E,F). All of the above data verified that NaHS attenuated hypoxia-induced cardiac fibroblast proliferation.

### 2.5. NaHS Represses Collagen Synthesis in Cardiac Fibroblasts with Hypoxia

**Figure 3:**
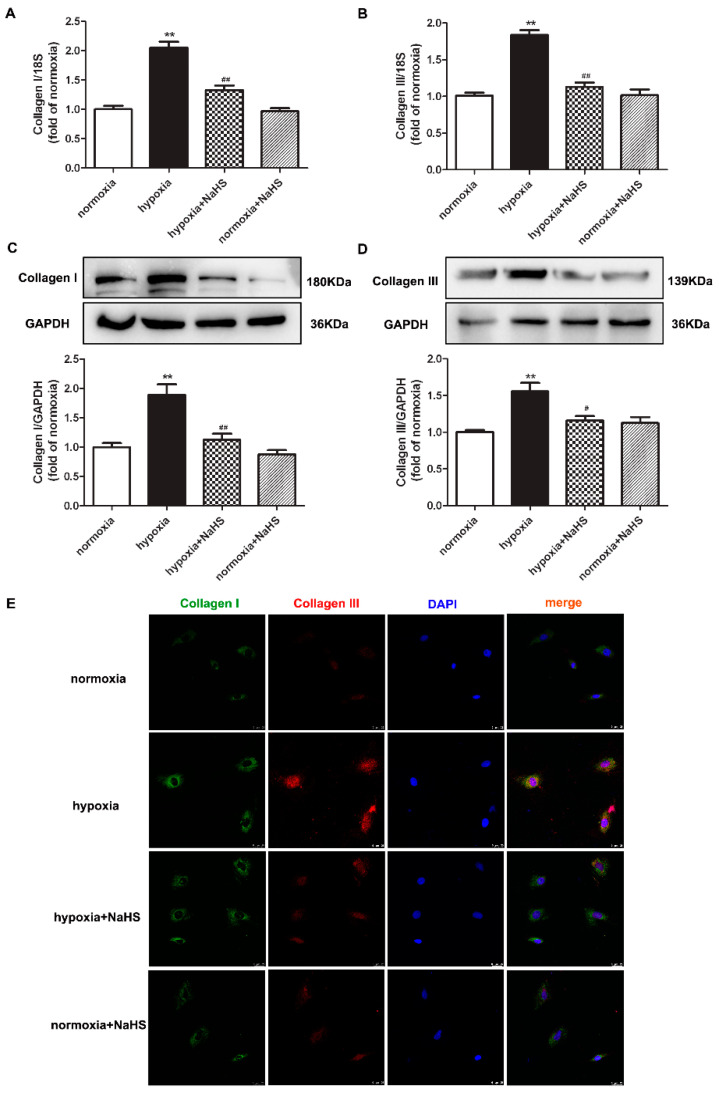
NaHS repressed the collagen synthesis in cardiac fibroblasts with hypoxia. After pre-administration with NaHS (50 μM) for 4 h, neonatal rat cardiac fibroblasts were subjected to normoxia (containing 5% CO_2_ and 95% O_2_) or hypoxia (containing 5% CO_2_ and 1% O_2_) for 4 h. (**A**,**B**) The collagen I and collagen III mRNA expression were detected by real-time PCR. (**C**,**D**) Collagen I and collagen III protein expression were detected by Western blot. ** *p* < 0.01 vs. normoxia; ^#^ *p* < 0.05, ^##^ *p* < 0.01 vs. hypoxia, *n* = 6. (**E**) Collagen I and collagen III were immunofluorescence stained with Alexa Fluor 488 (green) and Cy3 (red) conjugated IgG, respectively. The nuclei were stained with DAPI (blue). Bar = 25 μm.

Collagen type I and type III are the two main collagens synthesized by cardiac fibroblasts. Next, the expressions of the above two collagens were detected to evaluate the effect of NaHS on collagen synthesis in hypoxia-induced cardiac fibroblasts. Real-time PCR, Western blot, and immunofluorescence consistently verified that hypoxia increased both the collagen I and collagen III expression, which was significantly inhibited by NaHS pre-administration (Figure 3). These data suggested that NaHS repressed collagen synthesis in cardiac fibroblasts with hypoxia.

### 2.6. NaHS Restores DRP1 and OPA1 Protein Expression in Cardiac Fibroblasts with Hypoxia

**Figure 4:**
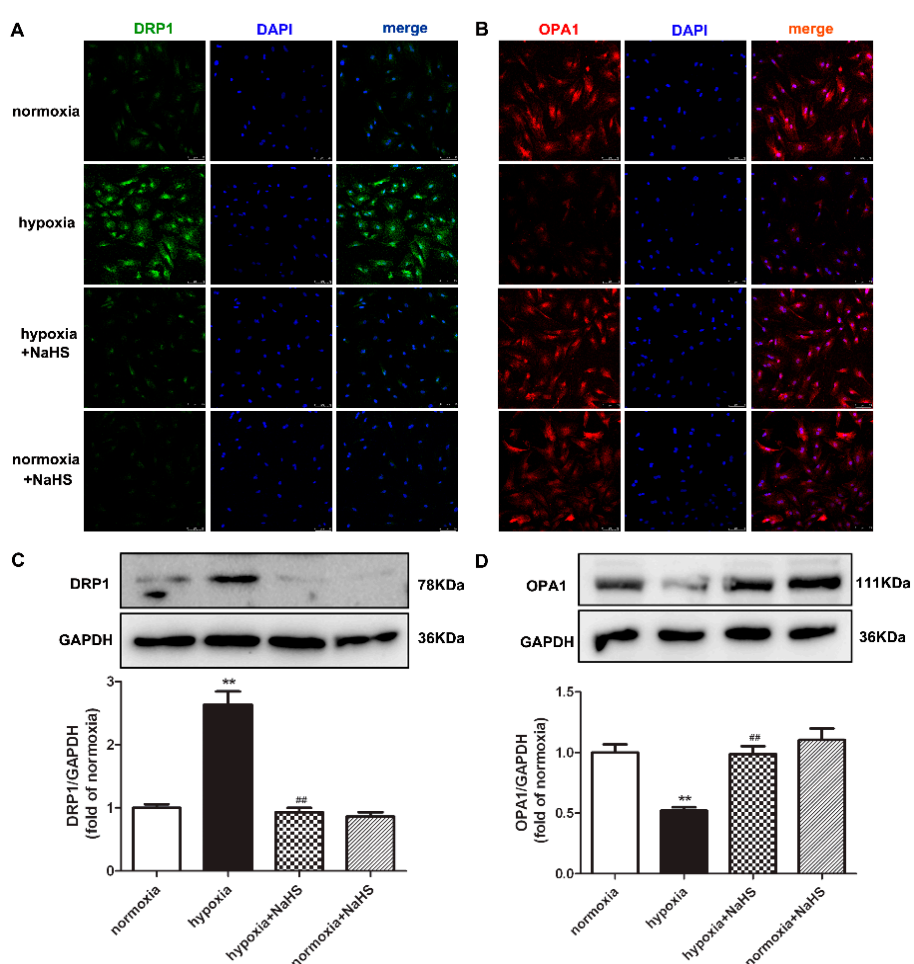
NaHS restored the DRP1 and OPA1 protein expression in cardiac fibroblasts with hypoxia. After pre-administration with NaHS (50 μM) for 4 h, neonatal rat cardiac fibroblasts were subjected to normoxia (containing 5% CO_2_ and 95% O_2_) or hypoxia (containing 5% CO_2_ and 1% O_2_) for 4 h. (**A**,**B**) DRP1 and OPA1 were immunofluorescence stained with Alexa Fluor 488 (green) and Cy3 (red) conjugated IgG, respectively. The nuclei were stained with DAPI (blue). Bar = 75 μm. (**C**,**D**) DRP1 and OPA1 protein expression were detected by Western blot. ** *p* < 0.01 vs. normoxia, ^##^ *p* < 0.01 vs. hypoxia, *n* = 6.

Dynamin-related protein 1 (DRP1) mainly mediates mitochondrial outer membrane fission, while optic atrophy 1 (OPA1) participates in mitochondrial inner membrane fusion. They both play a vital role in maintaining the mitochondrial structure [20]. Our present experiments found that hypoxia enhanced DRP1, but decreased OPA1 expression. Compared with hypoxia, NaHS pre-administration reduced DRP1 but elevated the OPA1 expression (Figure 4), suggesting that NaHS restored the balance between the DRP1 and OPA1 protein expression, which is beneficial to the mitochondrial homeostasis of fission and fusion in hypoxia-induced cardiac fibroblasts.

### 2.7. NaHS Alleviates Oxidative Stress in Cardiac Fibroblasts with Hypoxia

**Figure 5:**
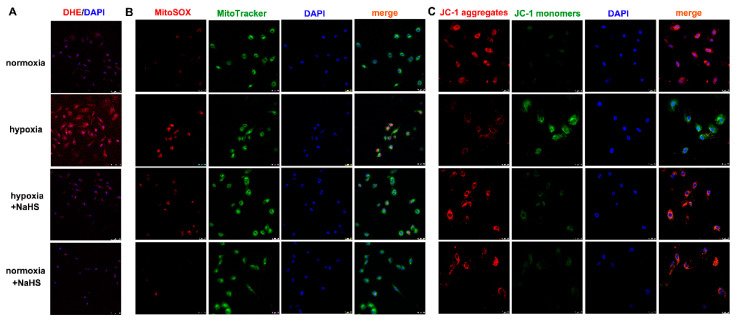
NaHS alleviated the oxidative stress and improved the mitochondrial membrane potential in cardiac fibroblasts with hypoxia. After pre-administration with NaHS (50 μM) for 4 h, neonatal rat cardiac fibroblasts were subjected to normoxia (containing 5% CO_2_ and 95% O_2_) or hypoxia (containing 5% CO_2_ and 1% O_2_) for 4 h. (**A**) The superoxide anion level was evaluated by DHE (red) staining. The nuclei were stained with DAPI (blue). Bar = 75 μm. (**B**) Mitochondrial ROS production was detected by MitoSOX (red) staining, and mitochondria were localized by MitoTracker (green) staining. The nuclei were stained with DAPI (blue). Bar = 25 μm. (**C**) The mitochondrial membrane potential was assessed by JC-1 staining. The nuclei were stained with DAPI (blue). Bar = 25 μm.

Dihydroethidium (DHE) easily entered living cells and incorporated into cell chromosomes to emit red fluorescence, which is commonly used to evaluate cellular superoxide anion. MitoSOX staining associated with mitochondrial co-localization is usually applied to detect ROS in the mitochondria. Our results showed that hypoxia strengthened the fluorescence intensity of DHE and MitoSOX staining, which was significantly attenuated by NaHS pre-administration (Figure 5A,B), suggesting that NaHS alleviated cellular and mitochondrial oxidative stress in cardiac fibroblasts with hypoxia.

### 2.8. NaHS Improves Mitochondrial Membrane Potential in Cardiac Fibroblasts with Hypoxia

Mitochondrial membrane potential impairment is a characteristic feature of early cell injury. Red fluorescence by JC-1 aggregates indicated normal mitochondria with a higher mitochondrial membrane potential, while green fluorescence by JC-1 monomers indicated impaired mitochondria with less mitochondrial membrane potential. Our JC-1 staining demonstrated that hypoxia decreased red, but enhanced green fluorescence. NaHS increased red but alleviated the green fluorescence of JC-1 staining (Figure 5C), suggesting that NaHS improved the mitochondrial membrane potential in cardiac fibroblasts with hypoxia.

### 2.9. NaHS Attenuates Necroptosis in Cardiac Fibroblasts with Hypoxia

**Figure 6:**
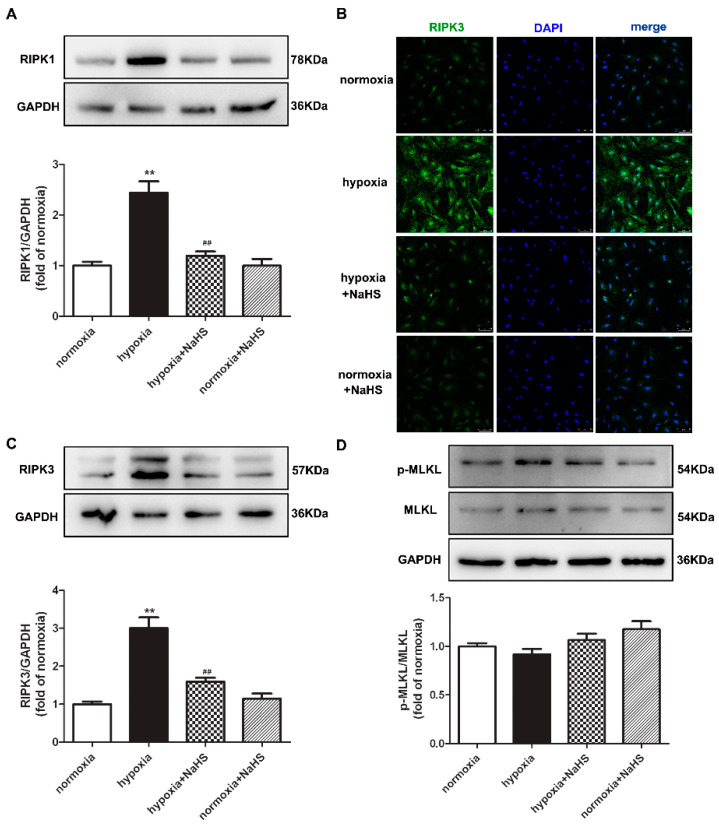
NaHS attenuated necroptosis in cardiac fibroblasts with hypoxia. After pre-administration with NaHS (50 μM) for 4 h, neonatal rat cardiac fibroblasts were subjected to normoxia (containing 5% CO_2_ and 95% O_2_) or hypoxia (containing 5% CO_2_ and 1% O_2_) for 4 h. (**A**) The RIPK1 protein expression was detected by Western blot. (**B**) RIPK3 was immunofluorescence stained with Alexa Fluor 488 (green) conjugated IgG. The nuclei were stained with DAPI (blue). Bar = 75 μm. (**C**,**D**) The RIPK3 and MLKL protein expressions were detected by Western blot. ** *p* < 0.01 vs. normoxia, ^##^ *p* < 0.01 vs. hypoxia, *n* = 6.

Previous studies have confirmed that RIPK1 and RIPK3 are characteristic indicators of necroptosis, and MLKL is one pathway of RIPK3 downstream to promote necroptosis [21]. The protein expressions of RIPK1, RIPK3, and MLKL in cardiac fibroblasts were detected by Western blot and/or immunofluorescence. This demonstrates that hypoxia increased the RIPK1 and RIPK3 expression, which was significantly alleviated by NaHS pre-administration (Figure 6A–C), suggesting that NaHS attenuated necroptosis in hypoxia-induced cardiac fibroblasts. However, there was no statistical difference in both the total and phosphorylated MLKL among each group (Figure 6D).

### 2.10. NaHS Enhances SIRT3 Expression in Cardiac Fibroblasts with Hypoxia

**Figure 7:**
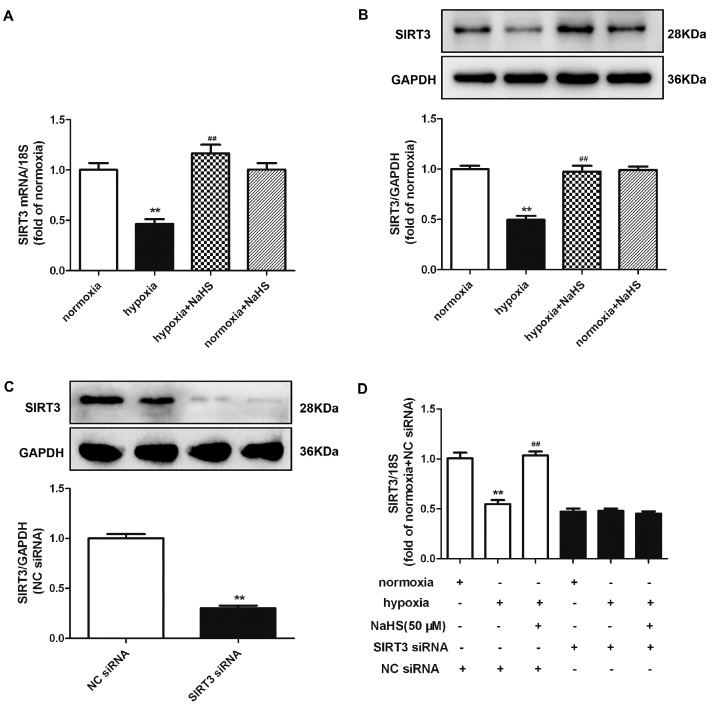
NaHS enhanced the SIRT3 expression in cardiac fibroblasts with hypoxia. (**A**,**B**) After pre-administration with NaHS (50 μM) for 4 h, neonatal rat cardiac fibroblasts were subjected to normoxia (containing 5% CO_2_ and 95% O_2_) or hypoxia (containing 5% CO_2_ and 1% O_2_) for 4 h. The SIRT3 mRNA and protein expression were detected by real-time PCR and Western blot, respectively. ** *p* < 0.01 vs. normoxia, ^##^ *p* < 0.01 vs. hypoxia, *n* = 6. (**C**) After SIRT3 siRNA or NC siRNA transfection with normoxia (containing 5% CO_2_ and 95% O_2_) for 48 h, the SIRT3 protein expression was detected by Western blot. ** *p* < 0.01 vs. NC siRNA. *n* = 6. (**D**) After transfection by SIRT3 siRNA or NC siRNA for 48 h, neonatal rat cardiac fibroblasts were pre-administrated with NaHS (50 μM) for 4 h followed by incubation with normoxia (containing 5% CO_2_ and 95% O_2_) or hypoxia (containing 5% CO_2_ and 1% O_2_) for another 4 h. The SIRT3 mRNA expression was detected by real-time PCR. ** *p* < 0.01 vs. normoxia-induced cells with NC siRNA transfection, ^##^ *p* < 0.01 vs. hypoxia-induced cells with NC siRNA transfection, *n* = 6.

Previous research verified that SIRT3 improved the mitochondrial dynamics by regulating the mitochondrial fission- and fusion-related proteins to exhibit cytoprotective effects [20]. Both real-time PCR and Western blot found that hypoxia significantly reduced the SIRT3 mRNA and protein expression, which was reserved by NaHS pre-administration (Figure 7A,B). However, whether NaHS inhibited cardiac fibroblast proliferation depending SIRT3 enhancement is unknown. Therefore, we aimed to explore the potential effects on hypoxia-induced cardiac fibroblasts by NaHS if SIRT3 was knocked down. After transfection, SIRT3 siRNA significantly decreased the SIRT3 expression in cardiac fibroblasts (Figure 7C). Moreover, NaHS enhanced the SIRT3 mRNA expression in hypoxia-induced cardiac fibroblasts after NC siRNA, but not SIRT3, transfection (Figure 7D).

### 2.11. NaHS Fails to Inhibit Cell Proliferation after SIRT3 Was Knockdown in Cardiac Fibroblasts with Hypoxia

**Figure 8:**
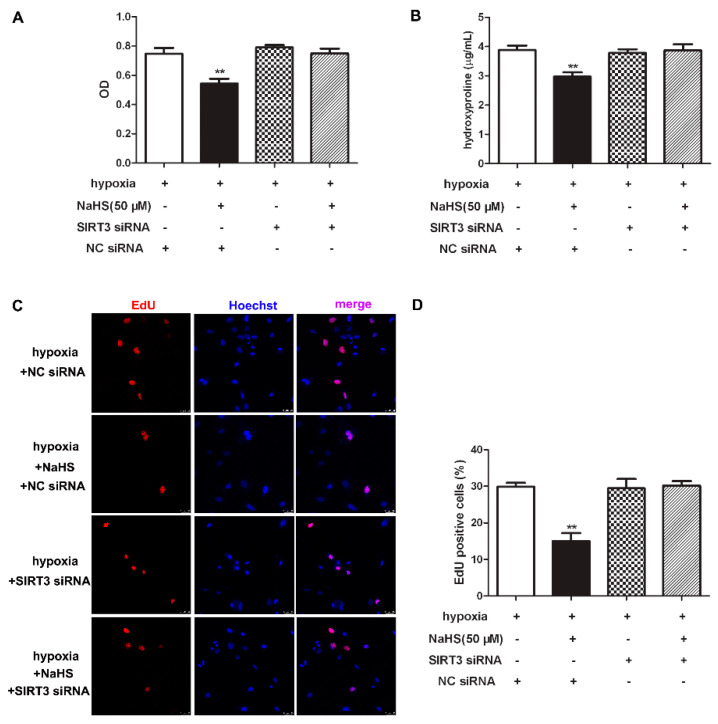
NaHS failed to inhibit cell proliferation after SIRT3 was knockdown in cardiac fibroblasts with hypoxia. After transfection by SIRT3 siRNA or NC siRNA for 48 h, neonatal rat cardiac fibroblasts were pre-administrated with NaHS (50 μM) for 4 h followed by incubation with normoxia (containing 5% CO_2_ and 95% O_2_) or hypoxia (containing 5% CO_2_ and 1% O_2_) for another 4 h. (**A**) The cardiac fibroblast number was measured with a CCK-8 kit. (**B**) The hydroxyproline content in the cell culture medium was detected. (**C**) Cell proliferation was assessed by EdU (red) staining. The nuclei were stained with Hoechst (blue). Bar = 25 μm. (**D**) The rate of EdU positive cells was quantified. ** *p* < 0.01 vs. hypoxia-induced cells with NC siRNA transfection, *n* = 6.

To clarify the role of SIRT3 of the inhibitory effects of NaHS on cell proliferation, CCK-8, hydroxyproline, and EdU staining was re-detected to explore the effect of NaHS on hypoxia-induced cardiac fibroblast proliferation after SIRT3 down-regulation. Our results showed that NaHS failed to decrease the cell number, reduce hydroxyproline content, or suppress the EdU positive ratio in hypoxia-induced cardiac fibroblasts after SIRT3 siNRA transfection (Figure 8). It suggested that the inhibitory effect of NaHS on hypoxia-induced cardiac fibroblast proliferation was unavailable after SIRT3 was knocked down.

### 2.12. NaHS Fails to Alleviate Mitochondrial Oxidative Stress and Attenuate Necroptosis after SIRT3 Was Knocked Down in Cardiac Fibroblasts with Hypoxia

**Figure 9:**
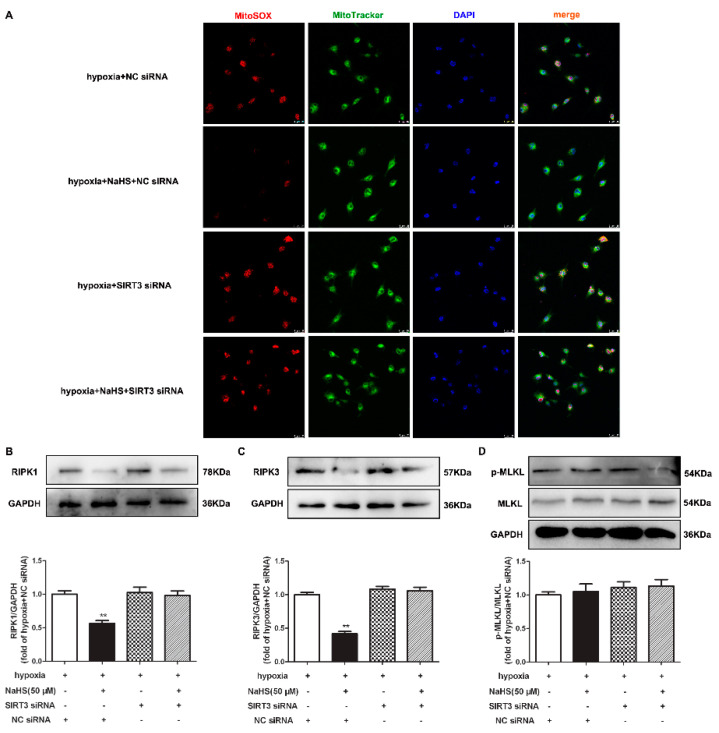
NaHS failed to alleviate mitochondrial oxidative stress and attenuate necroptosis after SIRT3 knockdown in cardiac fibroblasts with hypoxia. After transfection by SIRT3 siRNA or NC siRNA for 48 h, neonatal rat cardiac fibroblasts were pre-administrated with NaHS (50 μM) for 4 h followed by incubation with normoxia (containing 5% CO_2_ and 95% O_2_) or hypoxia (containing 5% CO_2_ and 1% O_2_) for another 4 h. (**A**) Mitochondrial ROS production was detected by MitoSOX (red) staining, and the mitochondria were localized by MitoTracker (green) staining. The nuclei were stained with DAPI (blue). Bar = 25 μm. (**B**–**D**) The RIPK1, RIPK3, and MLKL protein expressions were detected by Western blot. ** *p* < 0.01 vs. hypoxia-induced cells with NC siRNA transfection, *n* = 6.

Our present study also demonstrated that the inhibitory effects of NaHS on mitochondrial oxidative stress and RIPK1 or RIPK3 expression in hypoxia-induced cardiac fibroblasts disappeared after SIRT3 siNRA transfection (Figure 9A–C). However, there was no difference in the MLKL expression in each group (Figure 9D). All together, NaHS failed to alleviate mitochondrial oxidative stress and attenuate necroptosis after SIRT3 was knocked down in cardiac fibroblasts with hypoxia.

## 3. Discussion

Hypoxia as a result of insufficient physiological oxygen supply or excessive oxygen demand is the most common cause of cardiovascular diseases. Hypoxia usually results from absolute or relative insufficient oxygen supply to the myocardium with angina, acute myocardial infarction, and heart failure. Long term severe hypoxia can lead to excessive proliferation of cardiac fibroblasts and can impair cardiac function [22]. Our results showed that compared with the normoxia group; the cell numbers; hydroxyproline content; expression of sensitive markers for cell proliferation, including α-SMA, Ki67, PCNA, and EdU positive cells, were significantly increased, indicating that hypoxia successfully induced primary cardiac fibroblast proliferation, which effectively imitated the pathological properties of hypoxia-related myocardial fibrosis in vitro.

Previous studies have suggested that H_2_S alleviated the skeletal muscle fibrosis and partly improved skeletal muscle injury via oxidative stress inhibition [23]. Doxorubicin-induced myocardial fibrosis was significantly attenuated by H_2_S via suppressing ROS production [24]. H_2_S was also widely involved in renal fibrosis by ameliorating inflammation and oxidative stress [25]. All of these studies suggest that the anti-oxidant effect of H_2_S might be a common mechanism for benefits in different organ fibrosis. Indeed, ROS can act as a cellular signal to promote gene transcription and induce cell proliferation [26,27,28]. DHE and MitoSOX staining in our experiment confirmed that H_2_S alleviated oxidative stress, especially in the mitochondria of cardiac fibroblasts with hypoxia, may also be an important mechanism for cell proliferation inhibition.

The fission and fusion of mitochondria are collaboratively carried out with the participation of a variety of proteins. It is noteworthy that DRP1 and OPA1 affect mitochondrial division and fusion, respectively, in balance, to maintain mitochondrial structure and function [5]. Our experiment found that the expression of DRP1 was increased, while OPA1 was decreased in hypoxia-stimulated cardiac fibroblasts, indicating more fission in the mitochondria, which is a prerequisite for cell proliferation. On the other hand, the imbalance between DRP1 and OPA1 was prone to mitochondrial structure destruction and mitochondrial dysfunction, which ultimately promotes oxidative stress to accelerate cell proliferation. Moreover, NaHS pretreatment restored the equilibrium of DRP1 and OPA1, and corrected the imbalance of their expression, suggesting that the inhibitory effect of H_2_S on cardiac fibroblast proliferation with hypoxia by might be attributed to the correction of mitochondrial fission and fusion.

SIRT3, as an important deacetylase in mitochondria, regulates oxidative phosphorylation and energy metabolism through protein deacetylation [29,30]. The energy produced by mitochondria is mainly stored in the mitochondrial inner membrane. The mitochondrial membrane potential is formed due to the asymmetric distribution of the proton and ion concentration on both sides of the inner membrane [31]. Our study found that NaHS pretreatment significantly increased the mitochondrial membrane potential of the cardiac fibroblasts with hypoxia, which was closely related to maintaining the fission and fusion homeostasis of the mitochondrial membrane, and finally improved mitochondrial structure and function.

Moreover, previous studies demonstrated that SIRT3 deficiency promoted angiotensin II (Ang II)-induced cardiac fibrosis through ROS formation via transforming the growth factor β1 pathway [32]. SIRT3 knockout mice exacerbated cardiac fibrosis and diabetic cardiomyopathy [30,33]. In contrast, SIRT3 lentivirus transfection suppressed myofibroblasts transdifferentiation to attenuate Ang II-induced cardiac fibrosis via the signal transducer and activator of transcription 3 [34]. SIRT3 overexpression in cardiomyocytes also partially prevented tumor necrosis factor-α-induced inflammatory and profibrotic response [33]. Indeed, our present study verified that NaHS up-regulated the SIRT3 expression in cardiac fibroblasts with hypoxia stimulation, which may be involved in the inhibitory effects on cell proliferation and oxidative stress by H_2_S. In addition, the preventive effect of NaHS on mitochondrial oxidative stress and cell proliferation was greatly weakened after SIRT3 siRNA transfection, suggesting that SIRT3 may be a critical target for H_2_S to protect against hypoxia-induced cardiac fibrosis.

Necroptosis, as programmed cell death caused by ROS, is of great significance in a variety of pathophysiological processes, including development, tumor monitoring, homeostasis maintenance, and so on [15]. Some studies have found that SIRT3 regulated prostate cancer procession by suppressing the necroptosis-involved innate immune response [35]. Our data showed that both RIPK1 and RIPK3, as two sensitive markers for necroptosis, were increased in hypoxia-induced cardiac fibroblasts, which were suppressed by NaHS pre-treatment. Associated with the above anti-oxidant effects of NaHS, these results indicated that attenuated necroptosis might be related to the inhibitory effect of NaHS on oxidative stress. On the other hand, necroptosis inhibition is beneficial to alleviate cell damage, further weakening oxidative stress and forming a benign cycle. Moreover, after SIRT3 down-regulation, the inhibitory effect of NaHS on necroptosis was greatly weakened, which further confirmed that SIRT3 enhancement played a critical role in the inhibitory effect of NaHS on necroptosis. It is noteworthy that MLKL, a downstream substrate of RIPK3, remained unchanged in our present study. Similar to our previous studies where high glucose increased the RIPK1 and RIPK3, but not MLKL, expression in cardiomyocytes [36], the present study found that NaHS pretreatment of SIRT3 down-regulation did not alter the MLKL expression, suggesting that MLKL may not be involved in the process of hypoxia-induced necroptosis of cardiac fibroblasts, which might be related to pathological stimulation manners or cell injury degree. However, the potential pathways to mediate necroptosis in hypoxia-induced cardiac fibroblast proliferation are still unclear.

There are several limitations in our present study. Firstly, the third passages of the neonatal rat cardiac fibroblasts were used, meaning that these fibroblasts were fully activated and had a different phenotype than the resting fibroblasts in a normal healthy heart. Secondly, some other fibroblast functions, such as migration and/or contraction, as well as the degree of cell death, were not examined, which is beneficial to comprehensively evaluate the growth of cardiac fibroblasts. Thirdly, H_2_S promoted the chemical reprogramming of fibroblasts via metabolic rewiring [37]. However, little was known about metabolic reprogramming in the fibroblasts with hypoxia by H_2_S, which might be further examined in further studies.

**Figure 10:**
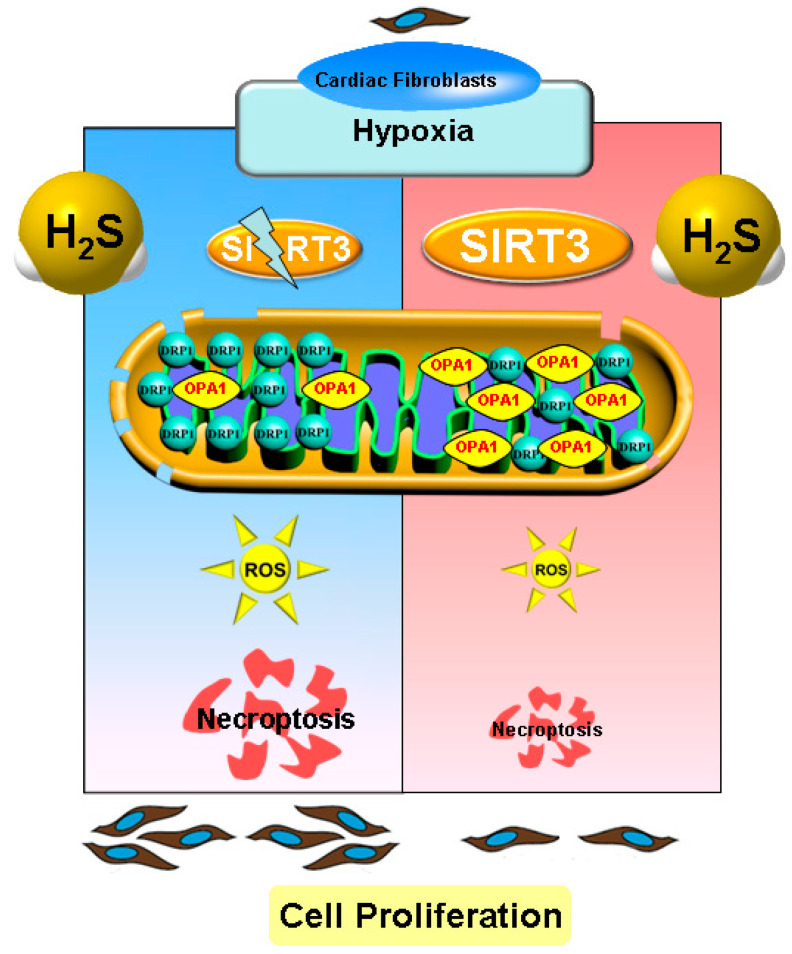
Illustration of the mechanism by which H_2_S suppressed hypoxia-induced cardiac fibroblasts proliferation. Exogenous H_2_S supplementation restored the balance between the DRP1 and OPA1 protein expression, reduced oxidative stress, attenuated necropoptosis, and inhibited cell proliferation in cardiac fibroblasts with hypoxia. H_2_S also enhanced SIRT3 expression in cardiac fibroblasts with hypoxia. Moreover, after SIRT3 siRNA transfection, the inhibitory effects of H_2_S on oxidative stress, necroptosis, and cardiac fibroblasts proliferation were all weakened. This suggests that necroptosis inhibition by H_2_S suppressed hypoxia-induced cardiac fibroblast proliferation via SIRT3.

In conclusion, necropoptosis inhibition by exogenous H_2_S supplementation alleviated cell proliferation, suppressed collagen synthesis, and reduced oxidative stress in cardiac fibroblasts with hypoxia, which was dependent on SIRT3 enhancement (Figure 10). Our study proposed a new idea for alleviating hypoxia-induced cardiac fibroblast proliferation, and provided a novel therapeutic strategy and biological target for the clinical prevention and treatment of myocardial fibrosis after hypoxia.

## 4. Materials and Methods

### 4.1. Primary Cardiac Fibroblasts Culture and Treatment

Newborn Sprague-Dawley (SD) rats with 3-day-old were anesthetized by isoflurane and were killed by decapitation. Then, the heart was quickly removed, cleaned, and cut into small pieces. Trypsin was added to digest the heart fragments for about 5 min in a water bath at 37 °C. Dulbecco’s modified eagle medium (DMEM, Wisent Inc., Montreal, QC, Canada) containing 10% fetal bovine serum (FBS, Wisent Inc, Montreal, QC, Canada) was timely added to stop excessive digestion. The above process was repeated 12–15 times. All of the digested supernatants, except the initial time, were collected for centrifugation. DMEM with 10% FBS was re-added to re-suspend the cell precipitate. After 4 h, the cardiac fibroblasts were mainly adhered onto the walls, while the cardiomyocytes were suspended in the medium that was abandoned by discarding the supernatant. Cardiac fibroblasts were sub-cultured and third passages were used in further experiments. After pre-administration with H_2_S donor sodium hydrosulfide (NaHS, 50 μM, Sigma-Aldrich, St. Louis, MO, USA) for 4 h, neonatal rat cardiac fibroblasts were subjected to normoxia (containing 5% CO_2_ and 95% O_2_) or hypoxia (containing 5% CO_2_ and 1% O_2_) for 4 h.

### 4.2. Measurement of H_2_S in the Culture Medium

The H_2_S concentration in the culture medium was measured using a H_2_S-specific microelectrode (ISO-H_2_S-2; World Precision Instruments, Sarasota, FL, USA) connected to a free radical analyser (TBR4100; World Precision Instruments), as previously described [4]. Briefly, the sensor was depolarized and calibrated with different concentrations of Na_2_S. According to the current change value and Na_2_S concentration, the standard curve was used to assess the H_2_S level in the culture medium.

### 4.3. siRNA Transfection

After starvation for 24 h, lipofectamine 3000 (Thermo Fisher Scientific Inc., Rockford, IL, USA) mixed with SIRT3 siRNA (5′-CCAUCUUUGAACUAGGCUUTT-3′ and 5′-AAGCCUAGUUCAAAGAUGGTT-3′, GenePharma, Shanghai, China) or negative control (NC) siRNA were added into the cell culture medium. After transfecting for 48 h, the SIRT3 protein expression was detected by Western blot to assess the efficiency of siRNA transfection. Then, cardiac fibroblasts were pre-administrated with NaHS (50 μM) for 4 h, followed by incubation with normoxia or hypoxia for another 4 h.

### 4.4. Cell Number Assay

After the above treatment, the CCK-8 working solution (Glpbio, Montclair, CA, USA) was added into the 96-well plate. After incubation at 37 °C for 1 h in the dark, the absorbance was measured at a wavelength of 450 nm. The relative optical density of each sample, normalized to the normoxia group, was representative of the cell number.

### 4.5. Hydroxyproline Content Determination

After treatment, the cell culture medium was collected to detect the hydroxyproline content using the introduction of the kits (Jiancheng Bioengineering Institute, Nanjing, China). The hydroxyproline content was calculated according to the standard concentration curve. The relative hydroxyproline content of each sample was normalized to the normoxia group.

### 4.6. EdU (5-Ethynyl-2′-deoxyuridine) Staining

After treatment, EdU (50 μM, RiboBio, Guangzhou, China) was added and incubated for 2 h. The cells were washed with PBS to eliminate the free EdU without incorporating it into DNA. Then, the cells were incubated with a fixative solution, glycine (2 mg/mL), and EdU penetrant in sequence. After Apollo staining, the reaction solution was stained at room temperature without light for 30 min, and the nuclei were stained with Hoechst 33342 for another 30 min. Red fluorescence by EdU and blue by Hoechst 33342 were observed and photographed with a laser confocal microscope (Leica, Wetzlar, Germany). EdU positive cells were quantified with Image J software.

### 4.7. JC-1 Staining

After treatment, the culture medium was discarded and PBS was added to wash the cardiac fibroblasts. The cells were incubated with a JC-1 working solution (Beyotime, Shanghai, China) in 37 °C without light for 20 min, followed by 4′,6-diamidino-2-phenylindole (DAPI) staining for 15 s. Red and green fluorescence by JC-1 were observed and photographed with a laser confocal microscope.

### 4.8. DHE Staining

After treatment, the cells were incubated with DHE (2 μM, Beyotime, Shanghai, China) at 37 °C without light for 30 min followed by DAPI staining for 15 s. The DHE fluorescence intensity, proportional to the superoxide anion level, was observed and photographed with a laser confocal microscope.

### 4.9. MitoSOX Staining

After treatment, cardiac fibroblasts were incubated with MitoTracker Green (200 nM, Beyotime, Shanghai, China) and MitoSOX (5 μM, Yeasen, Shanghai, China) in 37 °C without light for 30 min, followed by DAPI staining for 15 s. MitoSOX fluorescence intensity co-localized by MitoTracker Green, proportional to the mitochondrial reactive oxygen species (ROS) production, was observed and photographed with a laser confocal microscope.

### 4.10. Real-Time PCR

The total RNA was extracted from the cardiac fibroblasts with TRIzol. After reverse transcription, the cDNA was amplified by SYBR Green qPCR mixture (Takara, Otsu, Shiga, Japan) with real-time PCR systems (ABI 7500, Carlsbad, CA, USA). The primers were listed as: Collagen I, sense 5′-AGGGTCATCGTGGCTTCTCT-3′ and antisense 5′-CAGGCTCTTGAGGGTAGTGT-3′; Collagen III, sense 5′-AGCGGAGAATACTGGGTTGA-3′ and antisense 5′-GATGTAATGTTCTGGGAGGC-3′; SIRT3, sense 5′-GAGGTTCTTGCTGCATGTGGTTG-3′ and antisense 5′-AGTTTCCCGCTGCACAAGGTC-3′; 18S, sense 5′-AGTCCCTGCCCTTTGTACACA-3′ and antisense 5′-CGATCCGAGGGCCTCACTA-3′. The relative mRNA expressions of target genes were calculated based on 18S and were normalized as the fold of the normoxia group.

### 4.11. Western Blot

After treatment, the proteins were extracted from the myocardial fibroblast and the concentration was quantified using the BCA method. The proteins were boiled at 95 °C for 10 min and were stored in −80 °C. Then, the proteins were separated by sodium dodecyl sulfate-polyacrylamide gel electrophoresis (SDS-PAGE) and transferred to the polyvinylidene fluoride (PVDF) membranes. After the membranes were blocked with 5% non-fat milk for 2 h, diluted primary anti-α-SMA (1:1000), collagen I (1:500), collagen III (1:500) (Boster Biological Technology, Dublin, CA, USA); DRP1, OPA1, SIRT3, RIPK1, RIPK3, MLKL, p-MLKL (1:1000, Cell Signaling Technology, Danvers, MA, USA); and the GAPDH (1:5000, Sigma-Aldrich, St. Louis, MO, USA) antibody was added and incubated overnight at 4 °C. After washing, the secondary antibody (Beyotime, Shanghai, China) was incubated for 2 h at room temperature. The enhanced chemiluminescence (ECL, Thermo Fisher Scientific Inc., Rockford, IL, USA) solution was dropped onto the membrane to demonstrate the protein bands. The gray values of the bands were quantified using ImageJ software.

### 4.12. Immunofluorescence

After treatment and fixation, cardiac fibroblasts were incubated with blocking solution at room temperature. Then, diluted primary anti-α-SMA (1:100), collagen I (1:200), and collagen III (1:200) (Boster Biological Technology, Dublin, CA, USA); ki67 (1:100), PCNA (1:200) (ABclonal Technology, Wuhan, China); and the DRP1, OPA1, or RIPK3 (1:50, Cell Signaling Technology, Danvers, MA, USA) antibody were added and incubated overnight at 4 °C. After washing, the cells were incubated by IgG conjugated with Alexa Fluor 488 or Cy3 (1:500, Beyotime, Shanghai, China) without light at room temperature for 2 h followed by DAPI staining for 15 s. The cells were observed and photographed with a laser confocal microscope. The protein expression, which is considered as the fluorescence intensity, was quantified using ImageJ software.

### 4.13. Statistical Analysis

The experimental data were expressed as mean ± standard error (SEM), and analyzed by one-way analysis of variance followed by post-hoc test. The value of *p* less than 0.05 was considered to have statistical difference.

## Data Availability

All the data are available within the article.
